# Utility of *KRAS* Gene and Clinicopathological Features in the Assessment of the Risk of Type 2 Diabetes in the Etiology of Colon Cancer

**DOI:** 10.1055/s-0040-1714415

**Published:** 2020-08-31

**Authors:** Wedad Saeed Al-Qahtani, Ebtesam Al-Olayan, Fatimah Gh. Albani, Rania Saad Suliman, Nada Hamad Aljarba, E.M. Al-Humaidhi, Alanood S. Almurshedi, Dalia Mostafa Domiaty, Manal Abdullah Alduwish, Aljohara M. Al-Otaibi, Abdelbaset Mohamed Elasbali, Hussain Gadelkarim Ahmed, Bassam Ahmed Almutlaq

**Affiliations:** 1 Department of Forensic Sciences, Naif Arab University for Security Sciences, Riyadh, Saudi Arabia; 2 Department of Zoology, College of Science, King Saud University Riyadh, Riyadh, Saudi Arabia; 3 Department of Biology, College of Science, Princess Nourah bint Abdulrahman University, Riyadh, Saudi Arabia; 4 Department of Pharmaceutics, College of Pharmacy, King Saud University, Riyadh, Saudi Arabia; 5 Department of Biology, College of Science, Jeddah University, Jeddah, Saudi Arabia; 6 Department of Biology, Prince Sattam bin Abdulaziz University, College of Science and Humanities, Alkarj, Saudi Arabia; 7 Department of Clinical Laboratory Sciences, College of Applied Medical Sciences, Jouf University, Qurayyat, Saudi Arabia; 8 College of Medicine, University of Hail, Hail, Saudi Arabia; 9 Molecular Diagnostics and Personalized Therapeutics Unit, University of Ha'il, Hail, Saudi Arabia; 10 Department of Histopathology and Cytology, CMLS, University of Khartoum, Sudan

**Keywords:** colon cancer, KRAS, gene expression, immunohistochemistry, type 2 diabetes

## Abstract

**Background** Cancer and diabetes have a tremendous impact on health globally. This study aimed to evaluate the KRAS gene in colon cancer tissues obtained from patients with type 2 diabetes mellitus (T2DM).

**Materials and Methods** Data from 315 cases (156 colon diabetics and 159 patients were nondiabetics) were retrospectively retrieved. mRNA from surgically resected colon cancer tumors were also retrieved.

**Results** The expression of *KRAS* mRNA was significantly higher in patients afflicted with T2DM than nondiabetic patients. The *KRAS* mRNA levels were significantly amplified from primary to metastatic lesions ( *p* < 0.001).

**Conclusion** The association between T2DM and colon cancer was well-established in the present study.

## Introduction

Colon cancer and type 2 diabetes mellitus (T2DM) is an emerging health problem worldwide. 1 2 Both conditions share relatively similar risk factors, including, age, obesity, reduced physical activity, diet, smoking, and alcohol. 3 4 T2DM is often considered an independent risk factor for the progression of colorectal cancer. 5 The risk of colon cancer increases by 40 to 60% in patients with diabetes. 6 Although most of the risk factors for T2DM and colon cancers are similar, the potential epidemiological evidence linking the two is still lacking or not fully understood. One possible mechanism of cancer risk in diabetics is the elevated mitogenic activity due to hyperinsulinemia. This is attributed to high-insulin and IGF-1 that mediate the transformation and proliferation of colon cells, resulting in colon cancer. 7

One of the most common events in colorectal cancers is a mutation of *the KRAS* gene. *KRAS,* a member of the *RAS* gene family, is one of the most studied oncogenes present in the short arm of chromosome 12. Among the three human *RAS* genes, namely, *KRAS, NRAS* , and *HRAS, KRAS* is reported to be the most frequently mutated gene. 8 9 The *KRAS* gene encodes a 21kD *KRAS* protein involved in intracellular signal transduction processes. *KRAS* protein is activated upon binding with GTP, which is mediated by intracellular signals. Point mutation of *KRAS* at codon 12, 13, 59 or 61 impairs GTPase activity, thereby upregulating cellular proliferation and carcinoma progression. 10 About 20 to 50% of colorectal cancers are reported to be mutated *KRAS* gene, and the mutation frequency depends on the grade of the tumor. 11 This study aimed to evaluate the KRAS gene in colon cancer tissues obtained from patients with T2DM.

## Materials and Methods

In this study, 315 tissue samples of previously resected colon cancer tissues were retrieved from storage, including (a) fresh tissue specimens snap-freezing using liquid nitrogen, and stored at -80°C. (b) Formalin-fixed and paraffin-embedded (FFPE) tissue specimens. Fresh frozen tissues were used for molecular assessment. FFPE was used for conventional histopathology as well as immunohistochemistry (IHC) assessment. Of the 315 patients with colon cancer, 156 were diabetic patients (ascertained as cases) and the remaining 159 were nondiabetic patients.

Variables such as gender, age, size of the tumor, histological types, and other clinicopathological data were collected from their medical records.

### Analyses of KRAS Gene Expression

The first-strand cDNA was formed from the 2 µg of total RNA after utilizing random primers using the QuantiTect Reverse Transcription kit (Qiagen; Limburg, Netherlands) and 100 units/mL of reverse transcriptase, based on the protocol from the manufacturer. The utilization of primers for the cDNA amplification was developed after utilizing a web application (Primer3) founded on the sequences acquired from the National Center for Biotechnology Information (NCBI) database. The experiment was then normalized to GAPDH. qPCR or quantitative polymerase chain reaction was undertaken by using the SYBR Green PCR Core Reagent kit (Roche Diagnostics, Basel, Switzerland). At 95 °C for 10 minutes, the samples were denatured and amplified by 40 cycles (95 °C for 15 seconds), after which extension and annealing at 60 °C for 60 seconds was performed. The target gene amount relative to the reference gene GAPDH was quantified utilizing the cycle threshold (Cq). Amplification was then undertaken in duplicates with a real-time PCR system after utilizing the TaqMan reaction Master Mix (7500, Applied Biosystems, Grand Island, USA). The primer sequences included: *GADPH* , 5′-AACAGCCTCAAGATCATCAGCAA-3′ and 5′-CAGTCTGGGTGGCAGTGAT-3′; *KRAS* , 5′-CCTGCTGTGTCGAGAATATCCA-3′ and 5′-TTGACGATACAGCTAATTCAGAATCA-3′.

### Immunohistochemical Studies Using KRAS Antibody

*KRAS* protein expression of 79 metastatic tissue samples (49 from T2DM patients and 30 from nondiabetic patients) were evaluated by IHC. 12 Non-neoplastic colon mucosa adjacent to cancerous tissues served as an internal negative control. A 3-µm thick tissue sections were treated with 1:1000 dilution of *KRAS* antibody (F234 and SC-30) and automated stained using BenchMark ULTRA, based on the instructions of the manufacturer. The expression of the protein in at least 10% of tissue samples was scored as positive. Sections were visualized using Zeiss Axio Imager 2 research upright microscope. IHC scores were measured following standard procedure. 25

The KRAS was considered negative when it had scores of 0 and + 1and positive with scores of + 2 and + 3. To be considered as + 2 and + 3, the cell cytoplasm should be completely stained in more than 10% of the tumor cells. Cells without staining, with weak staining in part of the cell membrane, and in less than 10% of the tumor cells were considered negative.

### Statistical Analysis

The obtained data were analyzed via SPSS version 25. Pearson's correlation ( *r* ) was made to regulate the association of various clinicopathological variables and *KRAS* mRNA expression. Data were further validated using Akaike's information criterion, Hurvic, and Tsai's criterion. Variations in mRNA expressions between patients with and without T2DM and between primary and metastatic tumors were analyzed using one-way ANOVA. IHC scores (0–8) for each patient samples were summarized and analyzed using Pearson's correlation between non-neoplastic and metastatic tumor tissues; *p* values < 0.05 were considered as significant. Means were detached employing the Duncan Multiple Range Test (DMRT).

## Results

**Fig. 1:**
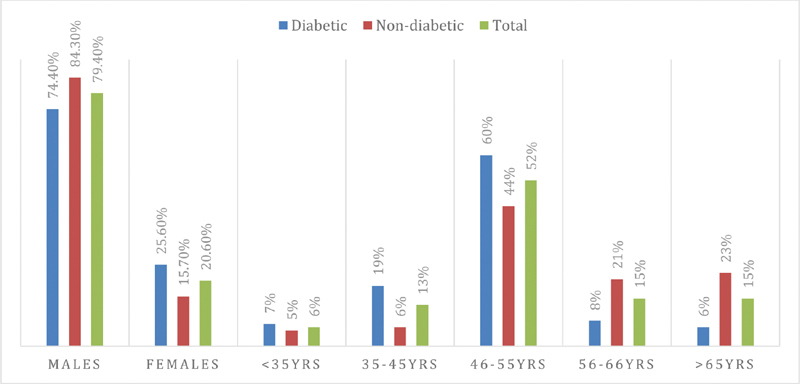
Description of colon cancer patients by sex and age.

Men represent the majority of patients with colon cancer 250/315 (79.4%), leading to a male female ratio of 1:00 to 3.85. Diabetic was common among females 40/65 (61.5%), hence most of the males' patients were nondiabetic 134/250 (53.6%). The majority of the patients were found in the age range 46–55 years, followed by 56–65 years, and > 65 years, representing 163/315 (51.7%), 47/315 (15%), and 46/3015 (14.6%), respectively. There was a relatively similar age distribution between diabetic and nondiabetic patients, as indicated in Table 1 and Fig. 1 .

**Table 1:** Distribution of colon cancer patients by sex and age

Variable	Diabetic	Nondiabetic	Total
**Sex**			
Males	116	134	250
Females	40	25	65
Total	156	159	315
**Age (years)**			
< 35	11	8	19
35–45	30	10	40
46–55	93	70	163
56–66	13	34	47
> 65	9	37	46

**Fig. 2:**
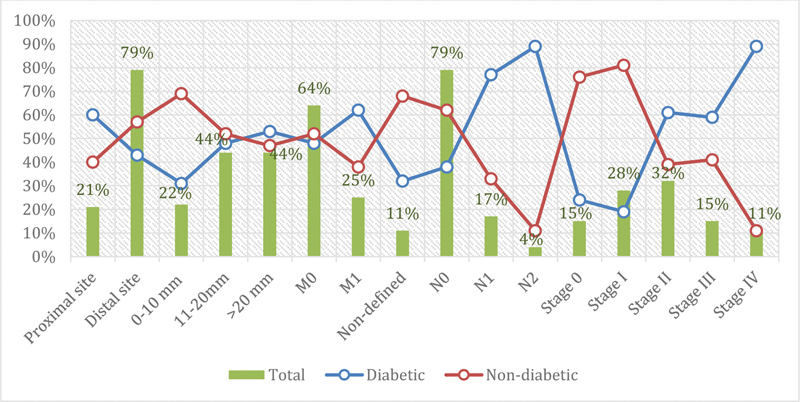
Description of colon cancer patients by clinicopathological features.

Table 2 and Fig. 2 , summarized the distribution of colon cancer patients by clinicopathological features. Concerning the lesion site, most lesions were in the distal colon, representing 197/315 (62.5%). The majority of the proximal site lesions were found among diabetic patients 32/53 (60.4%), whereas most distal site lesions were identified among nondiabetic patients 113/197 (57.4%). For tumor size, most patients were observed with a size range of 10 to 20 mm, representing 111/252 (44%). No immense difference in distribution between the diabetic and diabetic groups was observed in the tumor size, as indicated in Table 2 and Fig. 2 . About 79/315 (25%) of the tumors were metastasized (liver and rectal) as M1. A round 43/250 (17.2%) and 9/250 (3.6%) of the patients were found with N1 and N2 nodal involvement, in this order. Concerning the Union for International Cancer Control (UICC) staging, Stage II, Stage III, and Stage VI, were identified in 79/250 (31.6%), 37/250 (14.8%), and 27/250 (10.8%), as indicated in Table 2 and Fig. 2 .

**Table 2:** Distribution of colon cancer patients by clinicopathological features

Variable	Diabetic	Non-diabetic	Total
**Lesion site**			
Proximal colon	32	21	53
Distal colon	84	113	197
Total	116	134	250
**Tumor size (thickness in mm)**			
0–10 mm	17	38	55
11–20 mm	53	56	111
>20 mm	46	40	86
**Metastasis**			
M0	97	104	201
M1	49	30	79
Nondefined	10	28	38
**Nodal status**			
N0	75	123	198
N1	33	10	43
N2	8	1	9
**UICC stage**			
Stage 0	9	29	38
Stage I	13	56	69
Stage II	48	31	79
Stage III	22	15	37
Stage IV	24	3	27

Abbreviation: UICC, Union for International Cancer Control.

The *KRAS* expression was significantly complex in patients with T2DM than nondiabetics. Correlation between clinicopathological parameters showed a significant increase in expression, with an increase in the thickness ( *r* = 0.73, *p* = 0.02) and diameter ( *r* = 0.65, *p* = 0.03) of tumors in patients with T2DM. Similarly, increment in gene expression was noted with increase in tumor differentiation ( *r* = 0.54, *p* = 0.006) and depth of invasion ( *r* = 0.66, *p* = 0.001) among T2DM patients ( Table 3 ). Differential expression was observed between proximal and distal colon tumor sites in both the patient types. T2DM patients exhibited more mucinous tumors than nondiabetics. Expression was significantly upregulated in the metastatic tumor of both the patient types. The least information criterion of *KRAS* expression in a patient with T2DM was 713.721 ( *R* ^2^ = 0.53) based on the Akaike's information criterion, Hurvic, and Tsai's criterion.

**Table 3:** Correlations between *KRAS* gene expression and clinicopathological characteristics of patients with and without T2DM

Variables	With T2DM	Without T2DM
Tumor thickness	0.022 *	0.423
Tumor diameter	0.031 *	0.212
Tumor location	0.034 *	0.054
Tumor differentiation	0.006 *	0.065
Invasion depth	0.001 *	0.224
Mucin secretion	0.040 *	0.435
Metastasis	0.015 *	0.007 *
Lymph node status	0.320	0.765
Tumor stage	0.006 *	0.046 *

Abbreviation: T2DM, type 2 diabetes mellitus.

* Significant at *p* < 0.05.

**Fig. 3:**
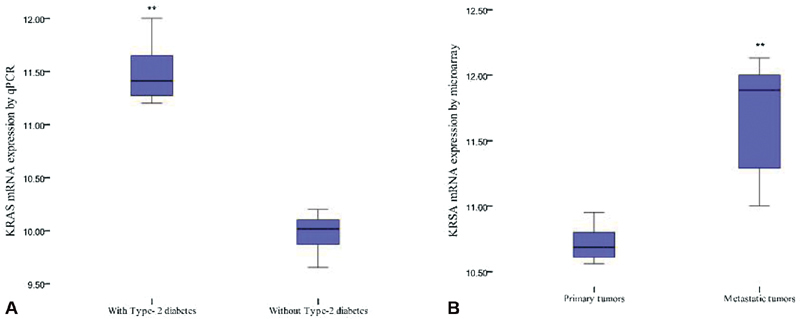
Box plots showing *KRAS* mRNA expression levels in patients ( **A** ) with or without type 2 diabetes mellitus (T2DM) and ( **B** ) between primary tumors and metastatic lesions. ** *p* <0.05.

Expression of *KRAS* mRNA in 156 cases with T2DM and 159 without T2DM is summarized in Fig. 3A . Elevated mRNA expression was significantly associated with the patient with T2DM and tumor stage. A two to six-fold increase in expression level was noticed in T2DM subjects than nondiabetics. Expression levels were normalized to *GADPH* mRNA. Expression of *KRAS* mRNA in 201 cases with primary tumors and 79 with metastatic lesions are summarized in Fig. 3B . The *KRAS* mRNA levels were significantly amplified from primary to metastatic lesions ( *p* < 0.001). The proportionate increase being 9.2% in primary tumors to 26.7% in metastatic lesions.

**Fig. 4:**
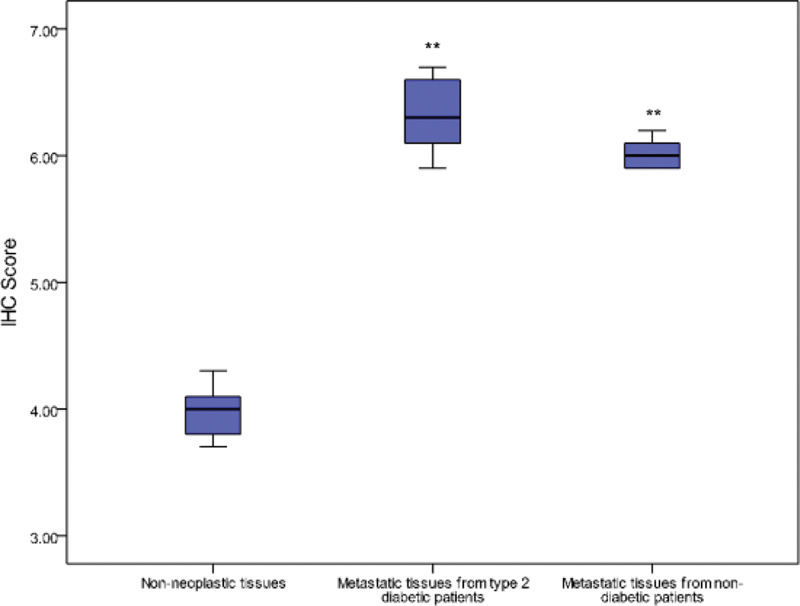
Immunohistochemical (IHC) scores for *KRAS* in non-neoplastic and metastatic tumor tissues among 79 patients. ** *p* < 0.05 versus normal.

*KRAS* protein expression was overexpressed in cancerous samples of T2DM patients. Two *KRAS* antibodies, F234 and SC-30, perform well in IHC on paraffin-embedded tissues. The results were more satisfactory for antibody F234, showing more positive scores compared with SC-30. IHC analyses showed positive cytoplasmic and nuclear staining. Immunohistochemical analyses indicated 56 to 83% *KRAS* protein expression in metastatic tumors from T2DM patients compared with 9 to 22% in nondiabetic patients. The upregulation of expression was associated with tumor invasion and advanced stage. The expression level exhibited different outcomes relative to tumor location. Expressions in metastatic tissues ( *r* = 0.79) were significant ( *p* < 0.05) compared with non-neoplastic tissues ( *r* = 0.48) collected adjacent to cancerous tissues. Average IHC scores for non-neoplastic colon tissues and metastatic tumor tissues ranged from 3.7 to 4.3 and 5.9 to 6.7, respectively for *KRAS* proteins ( Fig. 4 ).

## Discussion

Recent researches revealed an increasing trend of colorectal cancers in Saudi Arabia, most of which have been implicated in *KRAS* mutations. 13 Although *KRAS* mutations are highly restricted to colon cancer, it can be expressed in other cancers. 9 14

The present study found that patients with T2DM had higher colon cancer risk as revealed by elevated *KRAS* mRNA expression. These findings support the previous reports suggesting that patients with diabetes history have an increased risk for colorectal cancer. 15 A recent study reported a significant association between T2DM and the incidence of colorectal adenomas. This indicates that diabetic patients are at a higher risk of developing colorectal cancer, thus are in higher need for controlled colonoscopy. 16 17 However, these facts may suggest further considerations when assessing diabetic patients with colon cancer in terms of clinical features, treatment, and prognosis.

The current findings revealing a strong association between diabetic-related colon cancer and male sex. However, there is a lack of data in this context, but it might be attributed to an elevated number of colon cancer patients. The findings further indicate that diabetic patients with *KRAS* mutated gene had a higher risk of colon cancer compared with nondiabetics. This has an important clinical implication, as the management of T2DM might lower the risk of colon cancer. However, the results contradict some earlier studies, suggesting that diabetes does not play a role in the development of colon cancer. 18 Currently, epidemiological studies testified that diabetes intensifies the mortality rate of patients with colorectal cancers. 5

Further, the correlation between clinicopathological parameters showed a significant increase in *KRAS* mRNA expression, with an increase in thickness and diameter of tumors, tumor differentiation and depth of invasion in T2DM patients. Differential expression was observed between proximal and distal tumor sites. Proximal colon tumors were found to be associated with male, older age, advanced stage, and differentiated histology. Similar discrepancies were reported in earlier studies. 19

The findings of the present study might suggest that elevated *KRAS* mRNA expressions were associated with tumor-specific phenotypes. The weak expression in the patients without T2DM may be attributed to a lack of differentiated tumors. Such findings validate some of the earlier reports. 20 The *KRAS* mRNA expression was amplified significantly from primary to metastatic lesions. The present results were in agreement with some of the earlier literature on *KRAS* gene expression. 21 Besides, the association of *KRAS* mutation and diabetes with the risk of colon cancer, there might be other factors predisposing the development of colon cancer. Several explanations have been proposed for the increased risk of colon cancers in diabetic patients. These include prolonged bowel transit time, altered bile acid metabolism, hyperinsulinemia, and decreasing gut mucosa. 15 22 Immunohistochemical analyses indicated the upregulation of *KRAS* protein expression in metastatic tumors from T2DM patients with advanced-stage and tumor invasion. This may be attributed to the expression of the adhesion molecule promoting tumor invasiveness. 20 Expression in metastatic tissues was significant compared with adjacent non-neoplastic tissues. Furthermore, there is a lack of literature linking *KRAS gene* mutation to T2DM; thus, further studies to explore the association between *KRAS gene* mutation and T2DM would strengthen the genetic credibility of a cause-and-effect association via categorization of the molecular pathways incriminated. In other words, look for specific KRAS gene molecular signature associated with T2DM, and ultimately discover T2DM-specific approaches to prevent cancer-associated molecular evolution.

It was well-known that KRAS mutations in codons 12 and 13 are established predictive biomarkers for treatment of advanced colorectal cancer, with the antiepidermal growth factor receptor (EGFR) antibodies cetuximab and panitumumab. 23 24 KRAS involves mutations in several codons, some of which may show resistance to anti-EGFR treatment. However, this may further indicate the diversity in clinicopathological features, progression, and pattern of invasion and metastasis.

Although the present study provided valuable information regarding the association between T2DM and colon cancer or *KRAS gene* mutation in the Saudi population, it has some limitations including the absence of some variables such as the duration of T2DM and history of other comorbid conditions.

## Conclusion

The association between T2DM and colon cancer was well-established in the present study. Although *KRAS gene* mutation was related to colon cancer, the findings of this study suggest some intermolecular relationships with T2DM. Further search is needed to explore the interrelation between T2DM and *KRAS gene* mutation.
